# Organ and tumor dosimetry including method simplification for [^177^Lu]Lu-PSMA-I&T for treatment of metastatic castration resistant prostate cancer

**DOI:** 10.1186/s40658-024-00668-6

**Published:** 2024-07-17

**Authors:** Amir Karimzadeh, Linus Schatz, Markus Sauer, Ivayla Apostolova, Ralph Buchert, Susanne Klutmann, Wencke Lehnert

**Affiliations:** Department of Diagnostic and Interventional Radiology and Nuclear Medicine, University Medical Center Hamburg-Eppendorf, Martinistr. 52, 20246 Hamburg, Germany

**Keywords:** Radioligand therapy, Prostate cancer, ^177^Lu-PSMA, Dosimetry, Tumor dosimetry, [^177^Lu]Lu-PSMA-I&T, Single time point

## Abstract

**Background:**

Internal dosimetry in individual patients is essential for safe and effective radioligand therapy. Multiple time point imaging for accurate dosimetry is time consuming and hence can be demanding for nuclear medicine departments as well as patients. The objectives of this study were (1) to assess absorbed doses to organs at risk and tumor lesions for [^177^Lu]Lu-PSMA-I&T using whole body SPECT imaging and (2) to investigate possible simplified dosimetry protocols.

**Methods:**

This study included 16 patients each treated with 4 cycles of [^177^Lu]Lu-PSMA-I&T. They underwent quantitative whole body SPECT/CT imaging (3 bed positions) at four time points (TP) comprising 2 h, 24 h, 48 h and 72–168 h post-injection (p.i.). Full 3D dosimetry (reference method) was performed for all patients and dose cycles for organs at risk (kidneys, parotid glands and submandibular glands) and up to ten tumor lesions per patient (resulting in 90 lesions overall). The simplified dosimetry methods (SM) included (1) generating time activity curves for subsequent cycles using a single TP of imaging applying the kinetics of dose cycle 1, and for organs at risk also (2) simple extrapolation from dose cycle 1 and (3) from both, dose cycle 1 and 2.

**Results:**

Normalized absorbed doses were 0.71 ± 0.32 mGy/MBq, 0.28 ± 0.12 mGy/MBq and 0.22 ± 0.08 mGy/MBq for kidneys, parotid glands and submandibular glands, respectively. Tumor doses decreased from 3.86 ± 3.38 mGy/MBq in dose cycle 1 to 2.01 ± 2.65 mGy/MBq in dose cycle 4. Compared to the full dosimetry approach the SM 1 using single TP imaging at 48 h p.i. resulted in the most accurate and precise results for the organs at risk in terms of absorbed doses per cycle and total cumulated dose. For tumor lesions better results were achieved using the fourth TP (≥ 72 h p.i.).

**Conclusion:**

Simplification of safety dosimetry protocols is possible for [^177^Lu]Lu-PSMA-I&T therapy. If tumor dosimetry is of interest a later imaging TP (≥ 72 h p.i.) should be used/added to account for the slower kinetics of tumors compared to organs at risk.

**Supplementary Information:**

The online version contains supplementary material available at 10.1186/s40658-024-00668-6.

## Introduction

^177^Lu-labeled prostate-specific membrane antigen (PSMA) targeted radioligand therapy (RLT) has been acknowledged as an effective treatment for metastatic castration-resistant prostate cancer (mCRPC) due to its high efficacy and low toxicity [1–3]. A recent prospective phase III randomized clinical trial (VISION, NCT03511664) demonstrated prolonged overall and progression-free survival [4], resulting in the approval of [^177^Lu]Lu-PSMA-617 by the U.S. Food and Drug Administration and the European Medicines Agency. The corresponding dosimetry substudy reported a good safety profile and acceptable cumulated renal absorbed doses [5]. Dosimetry results for [^177^Lu]Lu-PSMA-617 have also been reported in [6–8] and reviewed in [9]. Due to inter-patient variability and in order to not compromise individual patient safety, some level of dosimetry should still be performed for such standardized treatment regimens. The European Council Directive 013/59/EURATOM, Article 56, mandates treatment verification [10], and qualitative verification at a suitable time point as well as safety dosimetry for organs at risk have been recommended [11–13]. Furthermore, a number of studies demonstrated positive correlations between baseline imaging parameters, absorbed radiation doses and treatment response [6, 14–16]. Therefore, patient-specific, dosimetry-guided treatment regimens that use higher activities or additional treatment cycles could be beneficial, leading to increased therapy efficacy while maintaining safety by keeping absorbed doses to organs at risk below predefined limits.

Internal dosimetry for RLT is generally performed based on the MIRD (Medical Internal Radiation Dose) formalism [17] using serial post-treatment imaging preferably with 3D quantitative SPECT (single photon emission computed tomography). However, multiple time point imaging can be restricted by scanner access and staff availability in nuclear medicine departments, and patient compliance during extended imaging sessions, especially in a patient group with partially heavily compromised health status [18]. A number of studies have indicated possible single time point image-guided dosimetry for organs at risk [19, 20] and tumor lesions [21–23].

Apart from [^177^Lu]Lu-PSMA-617, another low molecular weight PSMA ligand that has shown potential in treating mCRPC is [^177^Lu]Lu-PSMA-I&T, though clinical experience with it is still limited [24, 25]. It is currently being investigated in a prospective phase III randomized clinical trial for mCRPC patients who have undergone second line hormonal treatment but not chemotherapy (SPLASH, NCT04647526). Dosimetry data for [^177^Lu]Lu-PSMA-I&T are still limited and some are based on the more error-prone 2D planar imaging [26–28]. Two studies analysed both radiopharmaceuticals, [^177^Lu]Lu-PSMA-617 and [^177^Lu]Lu-PSMA-I&T, using identical dosimetry protocols applying 2.5D hybrid imaging [29] or 3D SPECT imaging [30] and reported favourable safety profiles for both [29] with some differences in terms of effective half-lives and absorbed doses [29, 30]. However, to the best of our knowledge, literature investigating the practicability of simplified dosimetry for [^177^Lu]Lu-PSMA-I&T is scarce [31, 32].

Therefore, the objectives of this retrospective analysis were:

- To assess absorbed doses and effective half-lives for organs at risk (kidneys, salivary glands, and submandibular glands) and tumor lesions in mCRPC patients who underwent at least four cycles of [^177^Lu]Lu-PSMA-I&T treatment using whole body quantitative SPECT/CT imaging,
- To investigate different simplified dosimetry methods using single time point imaging as well as simple dose extrapolations from the first dose cycle or first and second dose cycle in combination.

## Methods

### Patients

A total number of 16 patients have been analyzed (Table 1). All patients received at least 4 treatment cycles of [^177^Lu]Lu-PSMA-I&T (range 4–7). In total 72 treatment cycles were applied. Since all patients received at least 4 treatment cycles, dosimetry data was compared for this number of cycles to ensure consistency. Patients received an intravenous treatment with a median activity of 6.2 ± 0.5 GBq (range 5.0-6.7 GBq) [^177^Lu]Lu-PSMA-I&T for the first 4 cycles of treatment which could be slightly adopted based on e.g. lab test and tumor burden. Treatment was administred with a median interval of 8 weeks (range 6–10 weeks). Metastatic pattern was derived from baseline PSMA-ligand PET/CT imaging. At baseline pelvic lymph node, extrapelvic lymph node and bone metastases were present in 13 (81.3%), 11 (68.8%) and 16 (100%) patients, respectively.

**Table 1:** Patient characteristics

No of patients	16
Age (yr), median (IQR)	80 (77 − 4)
PSA (ng/ml), median (IQR)	56.4 (16.5-116.6)
LDH (U/l), median (IQR)	243.5 (187.8-284.3)
AP (U/l), median (IQR)	133.0 (83.5–193.0)
Hb (g/dl), median (IQR)	11.5 (10.2–12.6)
**Prior systemic therapies for mCRPC**,** n**	
Docetaxel	10
Cabazitaxel	4
Prior taxane-based chemotherapy	11
Abiraterone	7
Enzalutamide	9
Prior hormonal treatment with new hormonal agents	15
^223^Radium	1
**Site of metastasis**,** n**	
Lymph node pelvic	13
Lymph node extrapelvic	11
Bone overall	16

AP alkaline phosphatase, Hb haemoglobin, LDH lactate dehydrogenase, PSA prostate-specific antigen.

### SPECT/CT imaging

For each patient, whole-body SPECT/CT scans were acquired (3 bed positions from the eye socket to the upper thighs; 90 projections a’ 20 s, energy window: 208 keV ± 10%) on a Anyscan Trio SPECT/CT triple-head scanner (Mediso, Budapest, Hungary) at four time points (TP) at 2 h, 24 h, 48 h and 72 h to 168 h (mean 130 h) post-injection (p.i.). The scanner was equipped with a medium-low-energy general purpose (MLEGP) collimator. Quantitative image reconstruction was performed using the manufacturer’s Tera-Tomo™ 3D SPECT OSEM reconstruction with 110 effective iterations and 5 subsets applying CT-based attenuation correction, Monte Carlo-based scatter correction, and resolution recovery. To yield quantitative images (Bq/mL) a calibration factor was determined from an initial phantom experiment and automatically applied to each patient SPECT dataset.

### Dosimetry analysis

#### Reference method (RM)

Dosimetry analysis was performed for all patients and dose cycles using the QDOSE dosimetry software suite (ABX-CRO GmbH, Dresden, Germany). Organs at risk (kidneys, parotid glands and submandibular glands) and up to ten tumor lesions per patient were defined as source organs. Volumes of interest (VOIs) were segmented on the SPECT applying a flexible threshold to segment the predefined anatomical volumes of each organ/tumor and convolving this anatomical VOI with a Gaussian function (5 mm FWHM) to account for activity spill out. For dosimetry calculation the time integrated activities (TIA) were estimated using analytical integration to infinity based on a mono- or biexponential curve fit applied to the time activity curves (TACs). Trapezoidal integration was used where a fit to all data points was not possible due to long uptake phases, with an extrapolation to infinity using a monoexponential function fitted to the last 2 or 3 time points depending on when the excretion phase started. Dose calculation was performed using the phantom (kidneys) or spherical model (salivary glands and lesions) of IDAC-Dose 2.1 [33] considering only self-dose.

Individual kidney volumes were determined on contrast-enhanced CT scans and converted into masses for dose calculation using a density of 1.06 g/cm^3^. For a single parotid gland and submandibular gland ICRP organ masses of 25.0 g and 12.5 g were used, respectively [34]. Tumor lesions of interest were defined using pretherapeutic diagnostic PSMA-ligand PET/CT images selecting up to five target or measurable lesions according to RECIST and the five hottest lesions in the PET image, resulting in a maximum of ten lesions per patient. In total 90 tumor lesions consisting of 70 bone lesions, 19 lymph node lesions and 1 local recurrence were analyzed. Tumor volumetry was performed in the PET images using an adaptive threshold method [35, 36] in the software ROVER (ABX GmbH, Radeberg, Germany). Additionally, tumor masses were calculated with either a density of 1.03 g/cm^3^ for lymph node lesions or 1.92 g/cm^3^ (same as cortical bone) for bone lesions.

#### Simplified methods (SM)

The simplified dosimetry methods included the following three approaches:

- SM 1: generated TACs for cycles 2 to 4 using a single time point of imaging (at 2–4 h, 24 h, 48 h or 72–168 h) assuming the same pharmacokinetics as in dose cycle 1 and scaling the TAC accordingly. TIA and absorbed dose calculation were performed as for the RM. The absorbed dose D normalized to the injected activity was used for analysis.
- SM 2: was a simple extrapolation from the absorbed dose of the first cycle (D_1_), dividing it by the injected activity of the first cycle (A_1_) and multiplying it with the activity of the current i^th^ cycle (A_i_).

$$\:{D}_{i}=\frac{{D}_{1}}{{A}_{1}}\times\:{A}_{i}$$

- The total dose was then calculated over all 4 cycles.

- SM 3: is similar to SM 2. Instead of using the first cycle for extrapolation, D_i_ was calculated using the mean of the normalized absorbed doses from first (D_1_/A_1_) and second (D_2_/A_2_) cycle multiplied with the activity A_i_ of the current cycle.

$$\:{D}_{i}=\frac{\left(\frac{{D}_{1}}{{A}_{1}}+\frac{{D}_{2}}{{A}_{2}}\right)}{2}\times\:{A}_{i}$$

- The cumulated total dose over all 4 cycles was then calculated for further analysis.

### Statistical analysis

Normalized absorbed doses and effective half-lives were calculated for each patient and dose cycle using the reference dosimetry method. For comparison between dose cycles percent deviations from cycles 2 to 4 to the first cycle were calculated for each patient and as an average across patients for all organs and tumors. A mixed effects model test allowing for missing values was performed to analyze repeated measures data for mean normalized absorbed doses and mean effective half-lives between cycles for the RM.

Bland-Altman analysis [37, 38] was used to compare the simplified dosimetry methods SM 1, SM 2 and SM 3 to the reference method RM. For SM 1 this analysis was performed for the individual dose cycles 2, 3 and 4 and all cycles together. The different single imaging time points were considered separately. Lesion absorbed doses were analyzed individually across all patients and as mean absorbed dose per patient. Results are presented as relative difference (percent bias) and agreement limits (1.96 * standard deviation) which contain 95% of the data. In addition, the cumulated total absorbed doses over all dose cycles were compared between RM and SM 1, SM 2 and SM 3.

To further assess the accuracy of the SM compared to RM, the root mean square error (RMSE) was calculated as:

$$\:RMSE=\sqrt{\frac{{\sum\:}_{k=1}^{n}{\left({d}_{\:k,SM}-{d}_{k,RM}\right)}^{2}}{n}}$$

across n (all patients or all patients and dose cycles combined). Statistical analysis was carried out using GraphPad Prism Version 10.1.0 (264) for MAC.

## Results

### Normalized absorbed doses for organs at risk

**Fig. 1:**
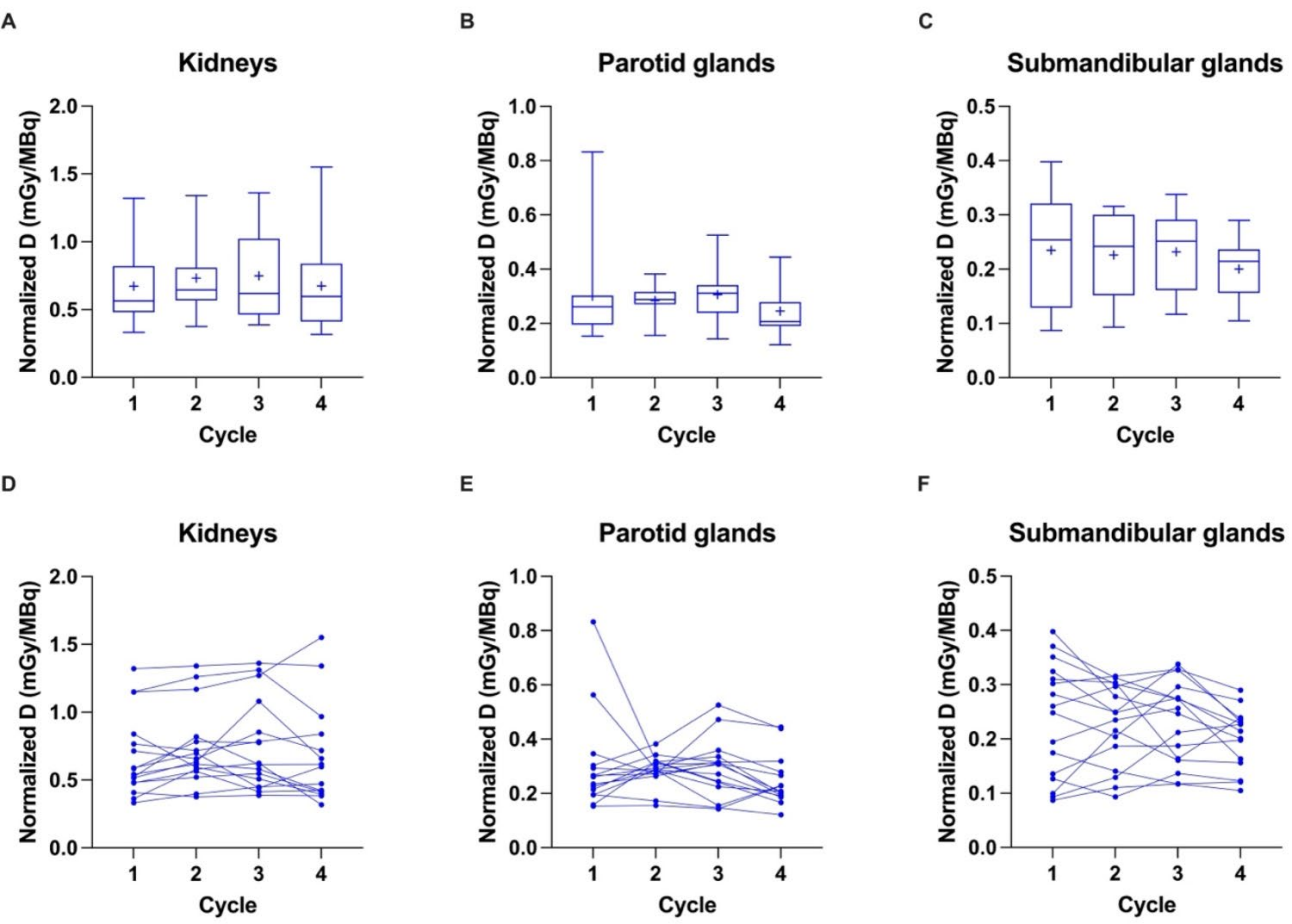
Box-Whisker-Plots showing the quartiles, the 5th and 95th percentiles (whiskers) and the mean (+) of the normalized absorbed doses (Normalized D) based on the RM across patients for kidneys (**A**), parotid glands (**B**) and submandibular glands (**C**). The course of values for each patient over the first four treatment cycles is shown for kidneys (**D**), parotid glands (**E**) and submandibular glands (**F**)

Mean normalized absorbed doses were highest for kidneys with 0.71 ± 0.32 mGy/MBq across dose cycles, respectively (Table 2; Fig. 1A). No significant difference was observed across the cycles (*p* = 0.19). This was also true for the parotid glands (*p* = 0.36) and submandibular glands (*p* = 0.22) with mean normalized absorbed doses of 0.28 ± 0.12 mGy/MBq and 0.22 ± 0.08 mGy/MBq across cycles, respectively (Table 2; Fig. 1B and C). The course of normalized absorbed doses for each patient is given in Fig. 1D-F.

**Table 2:** Normalized absorbed doses (mGy/MBq) based on the RM for organs at risk (kidneys, parotid glands and submandibular glands), individual tumor lesions (all, soft tissue lesions and bone lesions) and the mean of tumor lesions per patient (all, soft tissue lesions and bone lesions) for dose cycles 1 to 4. Data is presented as mean ± SD (range) including results of statistical analysis using a mixed effects model analyzing repeated measures

Cycle	Kidneys	Parotid glands	Submandibular glands
1	0.67 ± 0.30 (0.33–1.32); *n* = 16	0.30 ± 0.18 (0.15–0.83); *n* = 15	0.23 ± 0.11 (0.09–0.40); *n* = 16
2	0.73 ± 0.29 (0.38–1.34); *n* = 16	0.28 ± 0.06 (0.15–0.38); *n* = 16	0.23 ± 0.08 (0.09–0.32); *n* = 16
3	0.75 ± 0.33 (0.39–1.36); *n* = 16	0.31 ± 0.10 (0.14–0.53); *n* = 14	0.23 ± 0.08 (0.12–0.34); *n* = 16
4	0.67 ± 0.36 (0.32–1.55); *n* = 15	0.25 ± 0.09 (0.12–0.44); *n* = 15	0.20 ± 0.06 (0.12–0.29); *n* = 15
	*p* = 0.19	*p* = 0.36	*p* = 0.22
**Cycle**	**Individual tumor lesions (all)**	**Individual soft tissue lesions**	**Individual bone lesions**
1	3.86 ± 3.38 (0.06–16.08); *n* = 90	7.18 ± 3.94 (0.85–16.1); *n* = 20	2.91 ± 2.52 (0.06–11.2); *n* = 70
2	2.34 ± 1.88 (0.05–9.64); *n* = 87	3.98 ± 2.62 (0.33–9.64); *n* = 18	1.91 ± 1.37 (0.05–5.02); *n* = 69
3	2.04 ± 1.83 (0.11–10.6); *n* = 79	3.07 ± 3.13 (0.25–10.6); *n* = 18	1.73 ± 1.07 (0.11–4.78); *n* = 61
4	2.01 ± 2.65 (0.06–17.5); *n* = 67	3.36 ± 4.92 (0.16–17.5); *n* = 14	1.65 ± 1.50 (0.06–7.70); *n* = 53
	***p*** ** < 0.05**	***p*** ** < 0.05**	***p*** ** < 0.05**
**Cycle**	**Mean of tumor lesions per patient (all)**	**Mean of soft tissue lesions per patient**	**Mean of bone lesions per patient**
1	3.66 ± 2.81 (0.29–10.1)	5.37 ± 3.87 (0.85–12.5)	2.79 ± 2.38 (0.29–10.1)
2	2.18 ± 1.51 (0.17–4.69)	3.06 ± 2.34 (0.33–7.67)	1.83 ± 1.29 (0.17–4.03)
3	1.83 ± 1.29 (0.33–5.57)	2.34 ± 2.7 (0.25–8.26)	1.67 ± 0.94 (0.34–3.41)
4	1.70 ± 1.72 (0.17–7.15)	2.29 ± 3.72 (0.16–9.83)	1.62 ± 1.28 (0.18-5.00)
	***p*** ** < 0.05**	***p*** ** < 0.05**	***p*** ** < 0.05**

### Normalized absorbed doses for tumor lesions

**Fig. 2:**
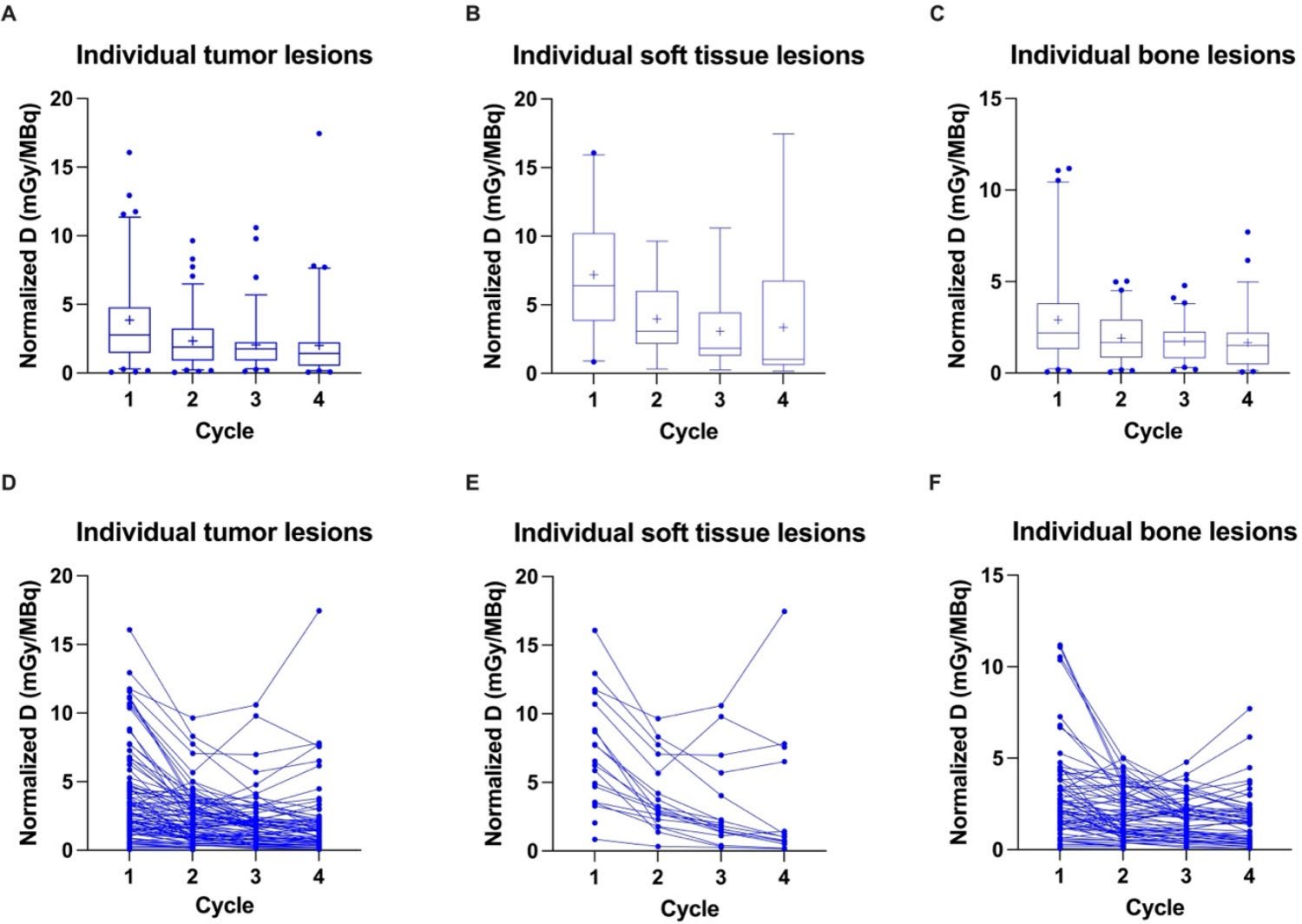
Box-Whisker-Plots showing the quartiles, the 5th and 95th percentiles (whiskers) and the mean (+) of the normalized absorbed doses (Normalized D) based on the RM across patients for individual tumor lesions (**A**), individual soft tissue lesions (**B**) and individual bone lesions (**C**) with outliers plotted as blue dots. The course of values over the first four treatment cycles is shown for individual tumor lesions (**D**), individual soft tissue lesions (**E**) and individual bone lesions (**F**)

Mean normalized absorbed doses for tumor lesions decreased from the first to the fourth treatment cycle (3.86 ± 3.38 mGy/MBq, 2.34 ± 1.88 mGy/MBq, 2.04 ± 1.83 mGy/MBq and 2.01 ± 2.65 mGy/MBq, respectively), showing a significant difference between cycles (*p < 0.05*; Table 2; Fig. 2A). This was also observed for soft tissue lesions (lymph node lesions and recurrent disease) and bone lesions (Table 2; Fig. 2B and C) when considered separately (both *p* < 0.05) but with higher normalized absorbed doses for soft tissue lesions compared to bone lesions across dose cycles. The course of values for each patient is given in Fig. 2D-F. Mean normalized absorbed doses of the mean of tumor lesions per patient and their stratification by soft tissue lesions and bone lesions are given in Table 2 and Supplementary Fig. S1.

### Effective half-lives

**Fig. 3:**
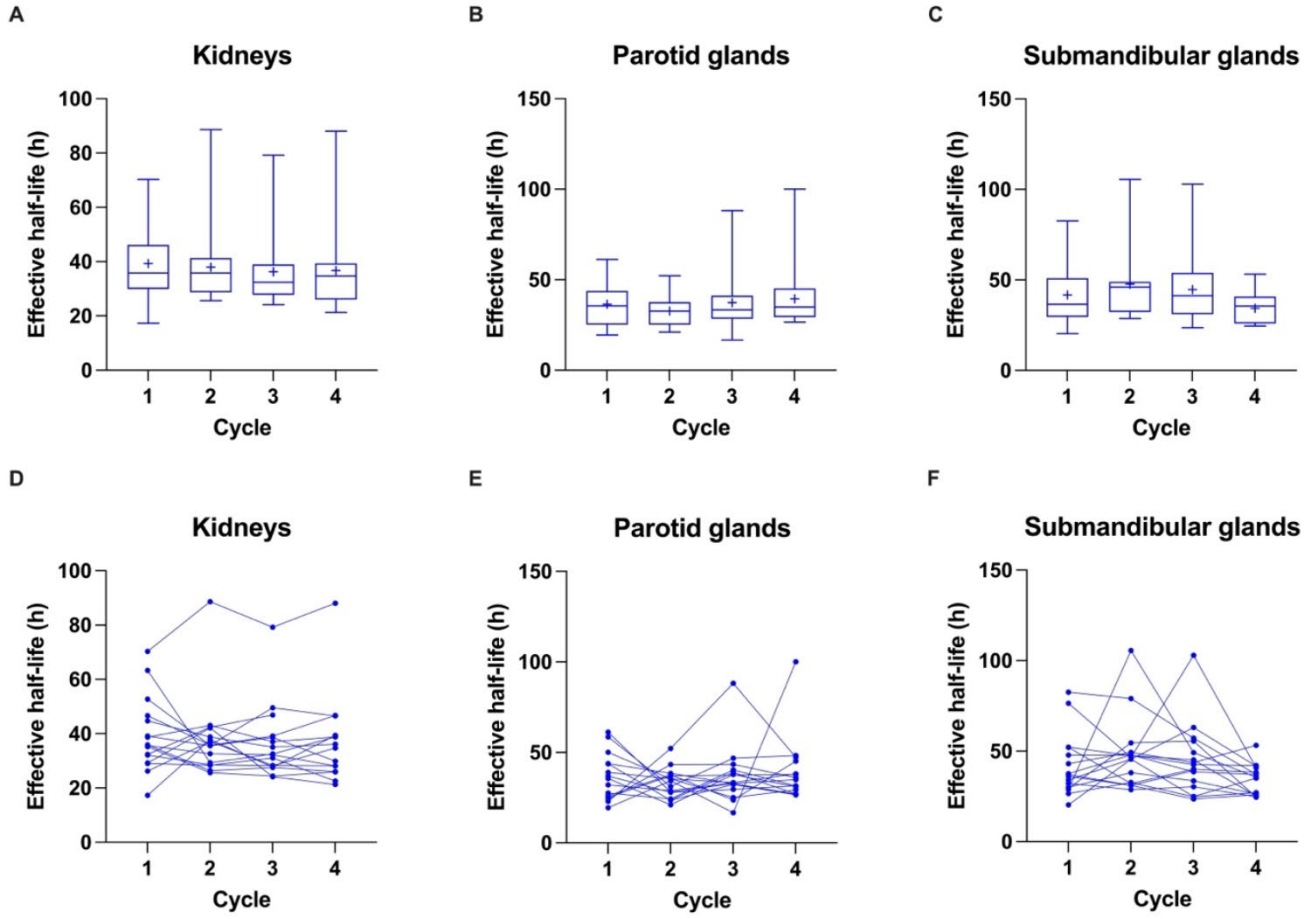
Box-Whisker-Plots showing the quartiles, the 5th and 95th percentiles (whiskers) and the mean (+) of effective half-lives based on the RM across patients for kidneys (**A**), parotid glands (**B**) and submandibular glands (**C**). The course of values over the first four treatment cycles is shown for kidneys (**D**), parotid glands (**E**) and submandibular glands (**F**)

**Fig. 4:**
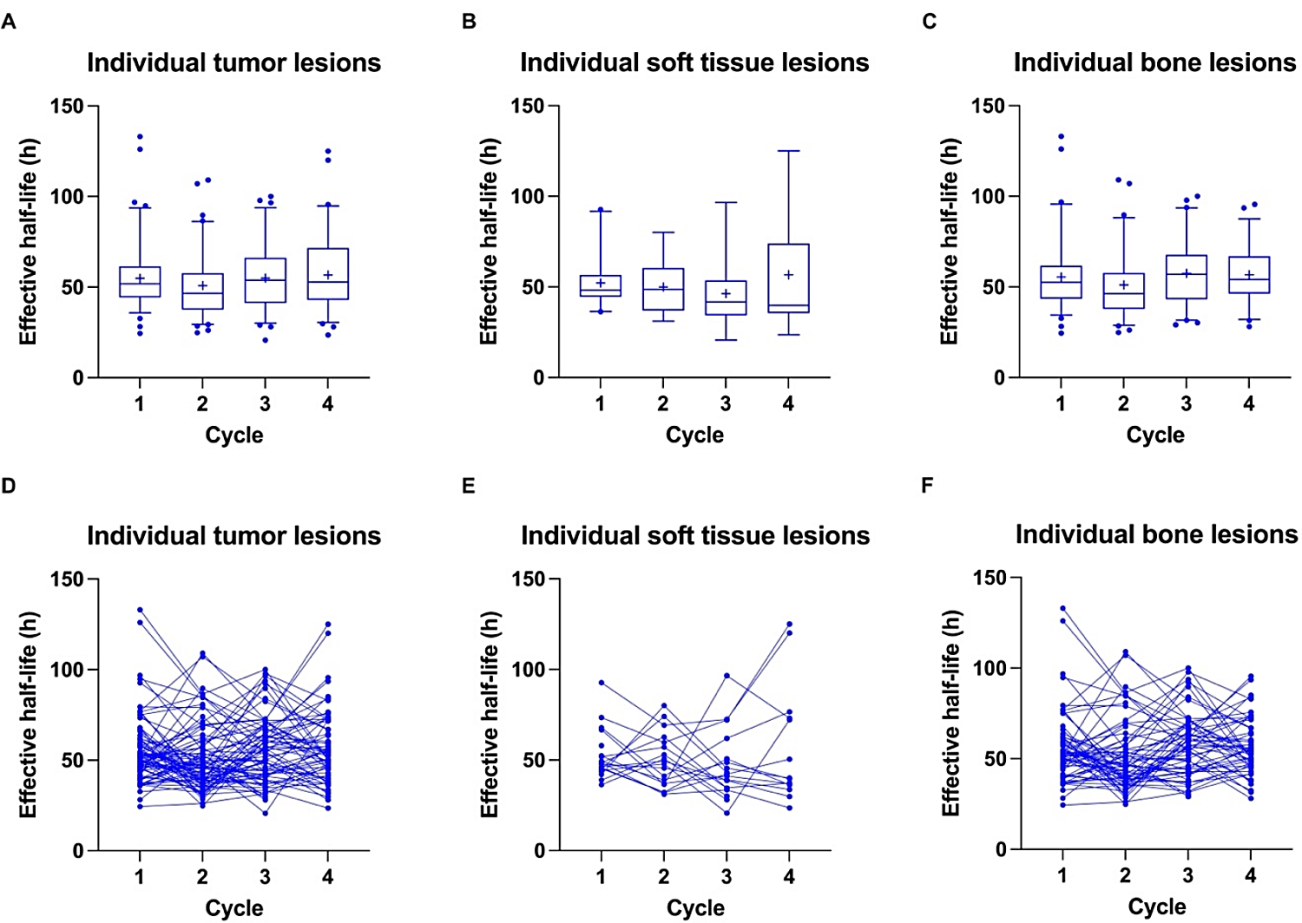
Box-Whisker-Plots showing the quartiles, the 5th and 95th percentiles (whiskers) and the mean (+) of effective half-lives based on the RM across patients for individual tumor lesions (**A**), individual soft tissue lesions (**B**) and individual bone lesions (**C**) with outliers plotted as blue dots. The course of values over the first four treatment cycles is shown for individual lesions (**D**), individual soft tissue lesions (**E**) and individual bone lesions (**F**)

Figures 3 and 4; Table 3 present the distribution of effective half-lives for organs at risk (kidneys, parotid glands and submandibular glands) as well as individual tumor lesions, individual soft tissue lesions and individual bone lesions based on the RM for cycles 1 to 4. Effective half-lives for the kidneys were 39.3 ± 13.7 h, 38.0 ± 14.6 h, 36.3 ± 13.6 h and 36.7 ± 16.3 h, respectively, with no significant difference between dose cycles (*p* = 0.58). For the parotid glands and submandibular glands they were 36.4 ± 13.0 h, 32.6 ± 8.2 h, 37.3 ± 16.7 h and 39.4 ± 18.2 h and 41.6 ± 17.3 h, 47.7 ± 19.8 h, 44.6 ± 19.5 h and 34.3 ± 8.5 h for dose cycles 1 to 4, respectively. Differences between effective half-lives were not significant for the partotid glands (*p* = 0.57) and for the submandibular glands (*p* = 0.07).

**Table 3:** Effective half-lives (h) based on the RM for organs at risk (kidneys, parotid glands and submandibular glands) and individual tumor lesions (all, soft tissue lesions and bone lesions) for dose cycles 1 to 4. Data is presented as mean ± SD (range) including results of statistical analysis using a mixed effects model analyzing repeated measures

Cycle	Kidneys	Parotid glands	Submandibular glands
1	39.3 ± 13.7 (17.3–70.3); *n* = 16	36.4 ± 13.0 (23.0-61.2); *n* = 15	41.6 ± 17.3 (26.6–82.5); *n* = 16
2	38.0 ± 14.6 (25.6–88.6); *n* = 16	32.6 ± 8.20 (21.1–52.2); *n* = 16	47.7 ± 19.8 (28.7-105.6); *n* = 16
3	36.3 ± 13.6 (24.1–79.2); *n* = 16	37.3 ± 16.7 (23.7–88.2); *n* = 14	44.6 ± 19.5 (23.6-102.9); *n* = 16
4	36.7 ± 16.3 (21.3–88.0); *n* = 15	39.4 ± 18.2 (26.6–48.3); *n* = 15	34.3 ± 8.50 (24.6–42.1); *n* = 15
	*p* = 0.58	*p* = 0.57	*p* = 0.07
**Cycle**	**Individual tumor lesions (all)**	**Individual soft tissue lesions**	**Individual bone lesions**
1	54.8 ± 18.1 (24.4–126.0); *n* = 90	52.2 ± 13.5 (36.3–92.7); *n* = 20	55.5 ± 19.2 (24.4–133.0); *n* = 70
2	50.8 ± 17.9 (24.8–109.0); *n* = 87	49.9 ± 14.8 (31.1–80.1); *n* = 18	51.1 ± 18.7 (24.8–109.0); *n* = 69
3	54.9 ± 18.1 (20.7–100.0); *n* = 79	46.3 ± 18.8 (20.7–96.5); *n* = 18	57.4 ± 17.3 (29.1–100.0); *n* = 61
4	56.7 ± 19.9 (23.5–125.0); *n* = 67	56.7 ± 32.5 (23.5–125.0); *n* = 14	56.7 ± 15.4 (28.0-95.6); *n* = 53
	*p* = 0.13	*p* = 0.36	*p* = 0.07

Compared to the organs at risk effective half-lives for individual tumor lesions were higher with 54.8 ± 18.1 h, 50.8 ± 17.9 h, 54.9 ± 18.1 h and 56.7 ± 19.9 h, respectively, but similar between dose cycles (*p* = 0.13) as opposed to the decrease in normalized absorbed doses. For individual soft tissue lesions and individual bone lesions effective half-lives were 52.2 ± 13.5 h, 49. 9 ± 14.8 h, 46.3 ± 18.8 h, 56.7 ± 32.5 h (*p* = 0.36) and 55.5 ± 19.2 h, 51.1 ± 18.7 h, 57.4 ± 17.3 h and 56.7 ± 15.4 (*p* = 0.07), respectively.

### Simplified dosimetry method 1 for organs at risk

**Fig. 5:**
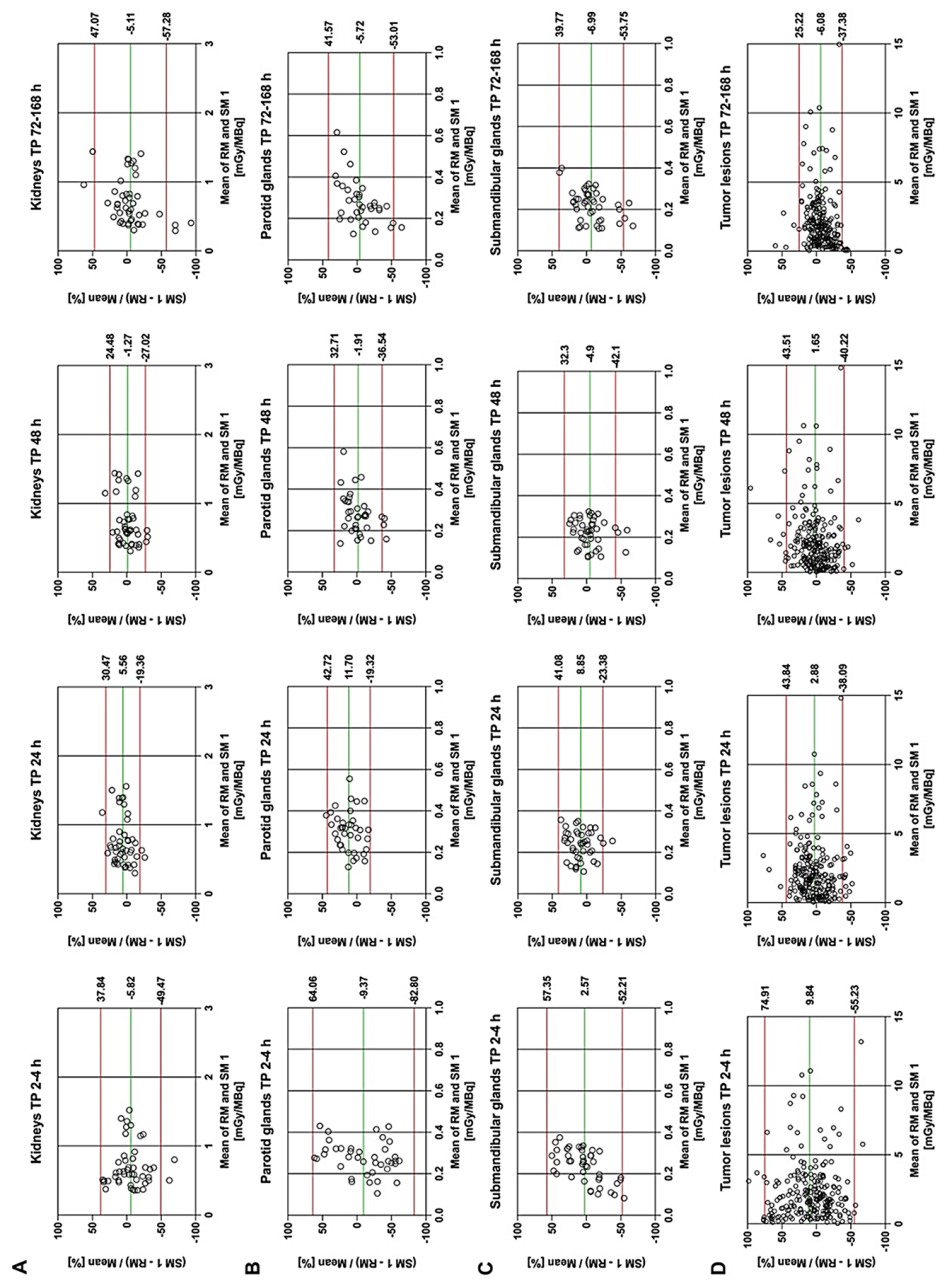
Bland-Altman plots for normalized absorbed doses comparing RM to SM1 using single time-point (TP) imaging at 2–4, 24, 48 and 72–168 h p.i. combined for treatment cycles 2 to 4 for kidneys (**A**), parotid glands (**B**), submandibular glands (**C**), individual tumor lesions (**D**). The green line represents the mean bias between the two methods, the red lines show the 95% limits of agreement

Figure 5A-C shows the Bland-Altman plots comparing the RM for organs at risk with the SM 1 using single TP imaging at four time points over all treatment cycles. Results of the Bland-Altman and the RMSE analyses are presented in Table 4. The average mean percent bias in kidneys absorbed dose was lowest at 48 h p.i., with − 1.27% and agreement limits of ± 25.8%. A slightly larger bias with a similar agreement limit was observed at 24 h p.i. with 5.56 ± 24.9%. For the parotid glands, the mean relative difference was lowest at 48 h p.i. (-1.91 ± 34.6%), while the smallest range of differences occurred at 24 h p.i. (11.7 ± 31.0%). Similarily, for the submandibular glands, bias and agreement limits were − 4.90 ± 37.2% at 48 h p.i. and 8.85 ± 32.2% at 24 h p.i. The RMSE was lowest for the TP at 48 h p.i. with 0.107 mGy/MBq, 0.048 mGy/MBq and 0.043 mGy/MBq for kidneys, parotid glands and submandibular glands, respectively. As outlined in Supplementary Tables S1 and Supplementary Figs. S2–S4, the Bland-Altman analysis of treatment cycles 2 to 4 considered separately showed similar results.

**Table 4:** Results of the bland-Altman analysis and RMSE comparing RM to SM1 using single time-point imaging at 2–4, 24, 48 and 72–168 h p.i. combined for treatment cycles 2 to 4 for organs at risk (kidneys, parotid glands and submandibular glands) and tumor lesions (individual tumor lesions and the mean of tumor lesions per patient)

Organ / structure	Method	Mean bias (%) ± 1.96*SD (min to max of 95% limits of agreement)	RMSE (mGy/MBq)
Kidneys	2–4 h p.i.	-5.82 ± 43.6 (-49.5-37.8)	0.146
	24 h p.i.	5.56 ± 24.9 (-19.4-30.5)	0.113
	48 h p.i.	-1.27 ± 25.8 (-27.0-24.5)	**0.107**
	72–168 h p.i.	-5.11 ± 52.2 (-57.3-47.1)	0.182
Parotid glands	2–4 h p.i.	-9.37 ± 73.4 (-82.8-64.1)	0.113
	24 h p.i.	11.7 ± 31.0 (-19.3-42.7)	0.065
	48 h p.i.	-1.91 ± 34.6 (-36.5-32.7)	**0.048**
	72–168 h p.i.	-5.72 ± 47.3 (-53.0-41.6)	0.085
Submandibular glands	2–4 h p.i.	2.57 ± 54.8 (-52.2-57.4)	0.066
	24 h p.i.	8.85 ± 32.2 (-23.4-41.1)	0.049
	48 h p.i.	-4.90 ± 37.2 (-42.1-32.3)	**0.043**
	72–168 h p.i.	-6.99 ± 46.8 (-53.8-39.8)	0.054
Individual tumor lesions	2–4 h p.i.	9.84 ± 65.1 (-55.2-74.9)	1.514
	24 h p.i.	2.88 ± 41.0 (-38.1-43.8)	1.488
	48 h p.i.	1.65 ± 41.9 (-40.2-43.5)	1.494
	72–168 h p.i.	-6.08 ± 31.3 (-37.4-25.2)	**1.431**
Mean of tumor lesions per patient	2–4 h p.i.	10.6 ± 49.6 (-39.0-60.2)	1.453
	24 h p.i.	4.24 ± 31.1 (-26.8-35.3)	1.412
	48 h p.i.	3.73 ± 30.3 (-26.6-34.0)	1.415
	72–168 h p.i.	-5.34 ± 24.7 (-30.0-19.3)	**1.350**

### Simplified dosimetry method 1 for tumor lesions

Results of the Bland-Altman and RMSE analyses comparing SM 1 to RM based on normalized absorbed doses for individual tumor lesions or the mean dose of tumor lesions per patient over all treatment cycles are displayed in Fig. 5D and Supplementary Fig. S5 and Table 4. For individual tumor lesions, the smallest range of dose differences was found at 72–168 h p.i. with a bias and agreement limit of -6.08 ± 31.3%. The mean relative difference in absorbed doses of tumor lesions was closest to zero at 48 h p.i., with a bias of 1.65 but a larger limit of agreement of ± 41.9%. Similar results were found considering the mean dose of tumor lesions per patient with the lowest relative difference at 48 h p.i. (3.73 ± 30.3%), while the smallest range of differences occurred at 72–168 h p.i. (-5.34 ± 24.7%). For tumor lesions the RMSE was lowest at TP 4 (72–168 h p.i.) with 1.431 mGy/MBq. In Supplementary Tables S2 and Supplementary Figs. S6 and S7, the Bland-Altman analysis of each treatment cycle demonstrated comparable results for individual tumor lesions and the mean of tumor lesions per patient.

### Total absorbed doses for simplified dosimetry methods 1, 2 and 3 for organs at risk

**Fig. 6:**
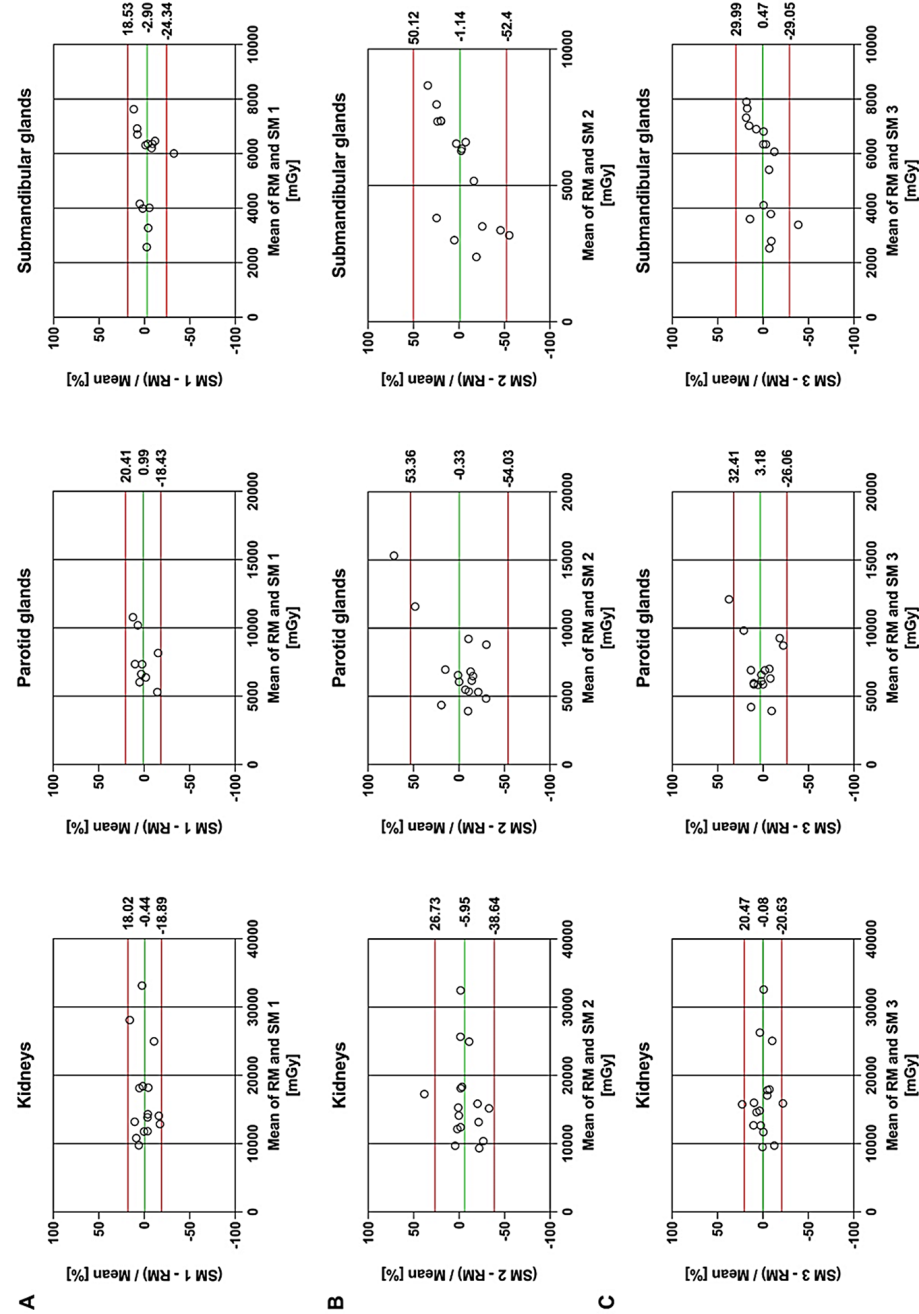
Bland-Altman plots for total cumulated absorbed dose over all 4 dose cycles for organs at risk comparing RM to SM1 (at 48 h p.i.; **A**), to SM 2 (**B**) and to SM 3 (**C**). The green line represents the mean bias between the two methods, the red lines show the 95% limits of agreement

Figure 6 shows the Bland-Altman plots comparing RM for organs at risk to SM 1, SM 2 and SM 3 in terms of total absorbed doses over 4 cycles. The highest precision was achieved with SM 1, using the favourable TP at 48 h p.i., with relative differences and limits of agreement for kidneys, parotid glands and submandibular glands of -0.44 ± 18.5%, 0.99 ± 19.4% and − 2.90 ± 21.4%, respectively. Comparing SM 2 and SM 3, better results were observed for SM 3 with relative differences and limits of agreement of -0.08 ± 20.5%, 3.18 ± 29.2%, 0.47 ± 29.5% for kidneys, parotid glands and submandibular glands versus SM 2 with − 5.95 ± 32.7%, -0.33 ± 53.7%, -1.14 ± 51.3%, respectively. Due to the observed decrease of normalized absorbed dose for tumors with subsequent dose cycles, SM 2 and SM 3 were not suitable to be applied to tumors.

## Discussion

### Absorbed doses and effective half-lives for [^177^Lu]Lu-PSMA-I&T

In our study, we have investigated dosimetry based on quantitative whole body SPECT/CT imaging for major organs at risk as well as tumor lesions for [^177^Lu]Lu-PSMA-I&T.

Mean normalized absorbed doses to the kidneys were with 0.71 ± 0.32 mGy/MBq (median 0.62 mGy/MBq) lower or similar to those reported for [^177^Lu]Lu-PSMA-I&T in the literature [26–29] (0.71–0.90 mGy/MBq) and similar or higher than those reported for [^177^Lu]Lu-PSMA-617 with 0.39–0.80 mGy/MBq [5–8, 19, 20, 23, 29]. Some of the differences of previously reported absorbed doses can potentially be attributed to differences in imaging methodology (planar vs. SPECT). Planar imaging has been shown to significantly overestimate absorbed doses to the kidneys for ^177^Lu-PRRT (peptide receptor radionuclide therapy) [39–42] and also in one study for [^177^Lu]Lu-PSMA-617 [23] but not in another study [43]. Furthermore, it has been shown previously that the tracer kinetics and absorbed doses for kidneys may differ between [^177^Lu]Lu-PSMA-I&T and [^177^Lu]Lu-PSMA-617 with higher absorbed doses caused by higher initial activity uptake found for [^177^Lu]Lu-PSMA-I&T [29, 30]. This is particularly important because a significant number of patients may experience moderate to severe declines in kidney function over the long term following the initiation of treatment [44]. The mean effective half-life was with 37.6 h also in the range of effective half-lives reported for [^177^Lu]Lu-PSMA-I&T with 33 h [29] and for [^177^Lu]Lu-PSMA-617 with 32.1–40.0 h [19, 22, 23, 29]. No differences in normalized absorbed doses and effective half-lives between therapy cycles were observed for the kidneys in our study while previously a small, but non-significant, increase has been reported for [^177^Lu]Lu-PSMA-617 between dose cycles 1 and 6 [19]. In two comparison studies longer effective half-lives were observed for [^177^Lu]Lu-PSMA-617 compared to [^177^Lu]Lu-PSMA-I&T with 40 h vs. 33 h [29] and 26 h vs. 20 h [30], respectively.

With regards to the salivary glands our observations with normalized absorbed doses of 0.28 ± 0.12 mGy/MBq for the parotid glands and 0.22 ± 0.08 mGy/MBq for the submandibular glands were lower compared to previous calculations with 0.50–1.30 mGy/MBq for [^177^Lu]Lu-PSMA-I&T [26–29] and 0.39–1.4 mGy/MBq for [^177^Lu]Lu-PSMA-617 [6–9, 19, 23, 29, 45] applying 2D planar or 3D SPECT imaging. One study also reported comparably low normalized absorbed doses to the salivary glands with 0.5 mGy/MBq for [^177^Lu]Lu-PSMA-617 using SPECT, while they demonstrated an overestimation of absorbed dose when planar imaging was used [23], but this was not confirmed in [43]. In addition, the observed differences may also be related to variability in methodology with respect to segmentation, organ mass and/or dose calculation. Mean effective half-lives with 36.4 h for the parotid glands and 42.1 h for the submandibular glands were, however, slightly larger than those shown previously for [^177^Lu]Lu-PSMA-I&T with 23 h [29] and 31–33.7 h [19, 23, 29] for [^177^Lu]Lu-PSMA-617. No significant differences in effective half-lives between dose cycles were seen for the salivary glands, as has also been shown in [19].

For tumors, mean absorbed doses for individual lesions were 3.86 ± 3.38 mGy/MBq with a higher mean of 7.18 ± 3.94 mGy/MBq for soft tissue lesions (lymph nodes + 1 recurrent disease) compared to bone lesions with 2.91 ± 2.52 mGy/MBq for dose cycle 1. The same trend could be shown for later dose cycles with mean absorbed doses in dose cycle 4 of 3.36 ± 4.92 mGy/MBq and 1.65 ± 1.50 mGy/MBq for soft tissue lesions and bone lesions, respectively. Between dose cycles a significant decrease of tumor dose was observed. Some lesions were not visible anymore in later dose cycles and hence had to be excluded from further analysis resulting in a decreasing number of analysed lesions across dose cycles. In general, mean tumor absorbed doses reported here for the first dose cycle were in line with those described previously of 3.20–5.80 mGy/MBq for [^177^Lu]Lu-PSMA-I&T [26–29] and 1.68–15.7 mGy/MBq for [^177^Lu]Lu-PSMA-617 as reviewed in [9] with high variability between individual lesions in each of the studies. Other studies also found a higher mean absorbed dose for lymph node lesions compared to bone lesions for [^177^Lu]Lu-PSMA-I&T [29] and [^177^Lu]Lu-PSMA-617 [29, 46]. However, opposing results were also reported for [^177^Lu]Lu-PSMA-617 [6–8, 47]. The effective half-lives were 54.3 h for all tumors, 51.3 h for soft tissue lesions and 55.2 h for bone lesions with no significant difference between cycles. Hence, changes in absorbed dose across dose cycles were solely based on reduced activity uptake in later cycles in response to the treatment and higher absorbed doses for soft tissue lesions compared to bone lesions were not caused by longer effective half-lives. Shorter or similar effective half-lives for tumors compared to this study were reported in the literature for [^177^Lu]Lu-PSMA-I&T with 43 h [29] and 51 h [48] while the values for [^177^Lu]Lu-PSMA-617 were somewhat larger with 61–69 h [22, 23, 29]. Although Schuchardt et al. also observed longer effective half-lives in a direct comparison study for [^177^Lu]Lu-PSMA-617 compared to [^177^Lu]Lu-PSMA-I&T with 61 h vs. 43 h, respectively, the median normalized absorbed doses were comparable for the two radioligands with 5.9 mGy/MBq vs. 5.8 mGy/MBq with [^177^Lu]Lu-PSMA-I&T exhibiting a higher initial uptake [29]. In our study no tumor sink effect was observed between cycles since a decrease in tumor dose or tumor burden did not result in an increase in kidneys dose.

Theoretically, the inconsistent last time point (inter- and partly intra-patient), a limitation of our study, could have led to differences in effective half-lives, i.e. potentially underestimating it with 72 h compared to 168 h. Even though some variability in effective half-lives between patients and/or dose cycles for individual patients (Figs. 3 and 4D-F) was observed in our study, this was, however, not found to be related to the different last time points. In general, comparisons for absorbed doses between different studies have to be done cautiously due to the differences in applied methodologies (e.g. planar imaging vs. SPECT, imaging time points, method of tumor/organ segmentation (activity and volume/mass), dose calculation approach).

Total cumulated absorbed doses after 4 dose cycles were in median 14.6 Gy with a minimum dose of 9.5 Gy and a maximum of 32.7 Gy for the kidneys, 6.7 Gy (4.1–10.1 Gy) for the parotid glands and 6.4 Gy (2.6–7.2 Gy) for the submandibular glands. These absorbed doses were below the proposed absorbed dose limits of 28–40 Gy for kidneys depending on risk factors and 35 Gy for salivary glands [49]. The kidneys were the dose limiting organ and not all patients could receive more than 4 dose cycles. Observing these dose limits for the kidneys is important as long-term nephrotoxicity has been shown for patients with higher renal dose beyond 28 Gy although no direct dose-response relationship was investigated [44]. Red bone marrow was not investigated in this study but should not be neglected as an organ at risk in clinical dosimetry.

### Simplified dosimetry methods

Implementing safety dosimetry would be desirable in routine clinical practice, as it contributes to ensuring individual patient safety. Single time point imaging in subsequent dose cycles after the first therapy should technically be possible for sites in Europe since a post-treatment scan is the most suitable option for the treatment verification after each therapy cycle required according to the European Council Directive 013/59/EURATOM Article 56 [6]. Comparable effective half-lives between dose cycles as also demonstrated in our study form the basis for this methodology. Single time point imaging has been more thoroughly studied for ^177^Lu-PRRT with varying suggestions for optimal time points between 24 h and 144 h for kidneys and/or tumors [48–58] but a number of studies have also investigated its use for [^177^Lu]Lu-PSMA-617 [19, 21–23].

In the current study, we compared the full dosimetry approach (RM) with the simplified method 1 (SM 1), which uses single time-point imaging. For organs at risk, the Bland-Altman analysis and RMSE (Root Mean Square Error) analysis revealed that the lowest mean percent bias (± limits of agreement) and RMSE were achieved using the third time point at 48 h p.i. For the kidneys, the bias was − 1.27 ± 25.8% (bias ± 1.96 SD, displayed for all data below for easier comparison between studies), and the RMSE was 0.107 mGy/MBq. For the parotid glands and submandibular glands, the bias and the RMSE were − 1.91 ± 34.6% and 0.048 mGy/MBq and − 4.90 ± 37.2% and 0.043 mGy/MBq, respectively. These findings are in line with those of Kurth et al. [19], who reported the smallest mean percent bias in absorbed doses and the smallest agreement limit in a Bland-Altman analysis using a single SPECT at 48 h after [^177^Lu]Lu-PSMA-617 treatment, with approximately 1.6 ± 17.8% for the kidneys and 0.6 ± 15.8% for the parotid glands considering six dose cycles [19]. Similar results were shown in a recently published analysis of 20 mCRPC patients treated with two cycles of [^177^Lu]Lu-PSMA-617, which also found that a single SPECT at 48 h p.i. was most suitable, based on the analysis of the kidneys with a percent bias compared to multiple time-point imaging of 4.6 ± 12.2% [22]. Another recent analysis for [^177^Lu]Lu-PSMA-617 based on two therapy cycles of 10 patients with hormone sensitive prostate cancer considered dosimetry using a single SPECT at 24–48 h p.i. feasible for kidneys and salivary glands [23]. Additional analysis for our study (not reported further) for the last time point considering only patients with a consistent TP 4 at 72 h (7 patients) did not result in an improvement for the organs at risk compared to single time point imaging at 48 h.

For individual tumor lesions and the mean of tumor lesions per patient the smallest range of dose differences were found at time point 4 (72–168 h p.i.) with bias and agreement limits of -6.08 ± 31.3% and − 5.34 ± 24.7%, respectively. In contrast, the mean relative differences were closer to zero at 48 h p.i. but with larger agreement limits (1.65 ± 41.9% and 3.73 ± 30.3%, respectively). However, for both, individual tumor lesions and the mean of tumor lesions per patient, the RMSE was lowest at 72–168 h p.i. This shows that, due to the slower pharmacokinetics of tumors, a later time point is preferable for a single time point image-based dosimetry approach in tumor lesions. These findings are in accordance with a recent study on [^177^Lu]Lu-PSMA-617 where SPECT imaging at 72 h p.i. exhibited the lowest agreement limits and a small bias compared to the multiple time-point approach with a bias and agreement limits of 3.7 ± 27.4% and 3.4 ± 17.4% (all: bias ± 1.96 SD) for individual lesions and whole tumor burden, respectively [22]. They have also shown that the simplification method proposed by Hänscheid et al. [50] provided similar results for their data [22]. Imaging time points beyond 72 h p.i. were not investigated in their study. Peters et al. who employed 5 time points at 1, 24, 48, 72 and 168 h p.i. deemed the latest time point as essential, while the most optimal simplified protocol included two imaging time points at 24 and 168 h p.i.for higher accuracy and lower uncertainty [23]. Another study used a population-based pharmacokinetic and found the most precise dose estimation using single time point imaging at 48–60 h p.i. for kidneys and salivary glands, and beyond 72 h p.i. for tumors [21]. We did not have a consistent late time point of imaging beyond 72 h p.i. and in this regard were not able to fully estimate an optimized imaging time point for tumor dosimetry.

Simplified dosimetry methods should be tested for each radiopharmaceutical of interest due to potential different pharmacokinetics for different radiopharmaceuticals. Despite the previously observed differences in the kinetics between [^177^Lu]Lu-PSMA-617 and [^177^Lu]Lu-PSMA-I&T [29, 30] our study showed that the results for single time point imaging for [^177^Lu]Lu-PSMA-617 are generally transferable to [^177^Lu]Lu-PSMA-I&T. There are two studies who investigated sampling schedules for [^177^Lu]Lu-PSMA-I&T [31, 32]. Rinscheid et al. [31] simulated TACs using a physiologically based pharmacokinetic (PBPK) model and biokinetic data of 13 patients. The single time point approach at 52 h p.i. using the method by Hänscheid [50] resulted in acceptable absorbed dose deviation (± SD) of -2.8 ± 6.4% for the kidneys, but did not lead to satisfactory results for tumor lesions [31]. Other simplification methods for extrapolation from the first cycle were not investigated in this study. Another study proposed a sampling scheme with imaging at days 1, 3 and 7 p.i. for kidneys and tumors, but did not examine single time point imaging [32].

Total cumulated absorbed doses for organs at risk over all 4 dose cycles were calculated using simple extrapolations from dose cycle 1 (SM 2) and dose cycles 1 and 2 (SM 3). SM 3 achieved similar results as SM 1 using single time point imaging at 48 h p.i. in terms of small bias with acceptable precision, in particular for the kidneys. SM 2 performed considerably worse and cannot be recommended when other options are feasible. Considerable underestimation of absorbed doses for kidneys and parotid glands were observed in [19] using the same approach resulting in a similar conclusion. Much improved precision was achieved in another study when every other dose cycle was included in the extrapolation [20]. Hermann et al. recommended a simple dose extrapolation from dose cycle 1 (as SM 2 in our study) based on the dosimetry substudy of the VISION trial, but the reference for this analysis was single time point imaging at 48 h p.i. from dose cycles 2–6 [5] and hence this reference might be biased itself. SM 2 and SM 3 cannot be applied to tumors due to the potential decrease in tumor dose with increasing dose cycle as observed in our study which is not reflected in these simplifications. In our study the observed accuracy and precision generally confirm the usability of the simplified methods SM 1 and for safety dosimetry also SM3 for routine dosimetry for [^177^Lu]Lu-PSMA-I&T in clinical departments. In any case, the individual patient condition should be taken into account and dosimetry results should be interpreted cautiously. Potential underestimation of absorbed dose using a simplified method could otherwise lead to exceeding critical cumulated absorbed doses and increase the risk of toxicity, e.g. in patients with impaired kidney function.

[^177^Lu]Lu-PSMA-I&T is currently investigated in a phase 3 trial (SPLASH, NCT04647526) and is expected to be approved for clinical use in the coming years. Hence the results of our study will hopefully contribute to clinical sites routinely employing dosimetry for [^177^Lu]Lu-PSMA-I&T in the future. Using simplified methods such as the single time point imaging technique (SM 1) at later dose cycles is beneficial for patients and clinical departments. It can relieve patients, in particular those in poor health condition and pain, from multiple distressing imaging sessions. Additionally, at sites where these treatments are performed on an outpatient basis without hospitalisation, as is common in North America, it has the logistical advantage that patients only have to return to the clinic once for imaging after therapy in subsequent dose cycles. For clinical departments it will hopefully increase the likelihood of individual patient dosimetry being performed in particular at sites with high patient numbers where these methods will lead to camera time being freed up for other examinations. Furthermore, single time point imaging, even though it has to be evaluated individually for each new radiopharmaceutical, should also contribute to a more rigorous use of dosimetry in clinical trials with adequate accuracy and precision at respective lower costs. This will hopefully help achieving the goal of individualising patient treatments for maximized efficacy.

## Conclusion

The absorbed doses reported here for [^177^Lu]Lu-PSMA-I&T were overall in the range of those reported previously for [^177^Lu]Lu-PSMA-617 and also [^177^Lu]Lu-PSMA-I&T for kidneys, salivary glands and tumors. In our study the kidneys were the major organ at risk.

For the prediction of absorbed dose using single time point imaging at 48 h p.i. starting from dose cycle 2 is feasible for safety dosimetry for [^177^Lu]Lu-PSMA-I&T. It would also be sufficient to extrapolate the dose for later dose cycles based on the injected activity and dosimetry results using full dosimetry imaging at dose cycles 1 and 2. Extrapolation from dose cycle 1 alone is possible but gave results with limited accuracy and should be applied with caution only if no other options are available.

For tumor dosimetry single time point imaging at or beyond 72 h p.i. may be possible for individual treatment planning, but further research using additional and consistent late time points would be needed to determine the most suitable time point. Simple extrapolations based on injected activity and dosimetry results at early dose cycles cannot be used for tumors due to the observed reduction of activity uptake and tumor dose in later dose cycles.

Altogether it was shown that simplified dosimetry methods are feasible for [^177^Lu]Lu-PSMA-I&T with the capacity of reducing the burden on clinical departments and patients while allowing for safe and potentially more effective radioligand therapies for individual patients.

### Electronic supplementary material

Below is the link to the electronic supplementary material.

Supplementary Material 1

## Data Availability

The datasets supporting the conclusions of this study can be made available on reasonable request.

## Abbreviations

2D, 2.5D, 3D: 2, 2.5 and 3-dimensional
[^177^Lu]Lu-PSMA-I&T: ^177^ Lutetium-labeld prostate-specific membrane antigen
AP: Alkaline phosphatase
CT: Computed tomography
FWHM: Full width at half maximum
Hb: Haemoglobin
LDH: Lactate dehydrogenase
mCRPC: Metastatic castration-resistant prostate cancer
MLEGP: Medium low energy general purpose
OSEM: Ordered subset expectation maximization
PBPK: Physiological based pharmacokinetic modeling
PET: Positron emission tomography
p.i.: Post injection
PSA: Prostate-specific antigen
RECIST: Response evaluation criteria in solid tumours
RLT: Radioligand therapy
RM: Reference method
RMSE: Root mean square error
SD: Standard deviation
SM: Simplified method
SPECT: Single-photon emission computed tomography
TAC: Time activity curves
TIA: Time integrated activity
TP: Time point
