# Supplementary material for: Organ and tumor dosimetry including method simplification for [177Lu]Lu-PSMA-I&T for treatment of metastatic castration resistant prostate cancer
Source: EJNMMI Phys. 2024 Jul 17;11:63. doi: 10.1186/s40658-024-00668-6 (PMC11255161; doi:10.1186/s40658-024-00668-6)
Supplement: Supplementary file 1 — Supplementary Material 1 [file 40658_2024_668_MOESM1_ESM.docx]

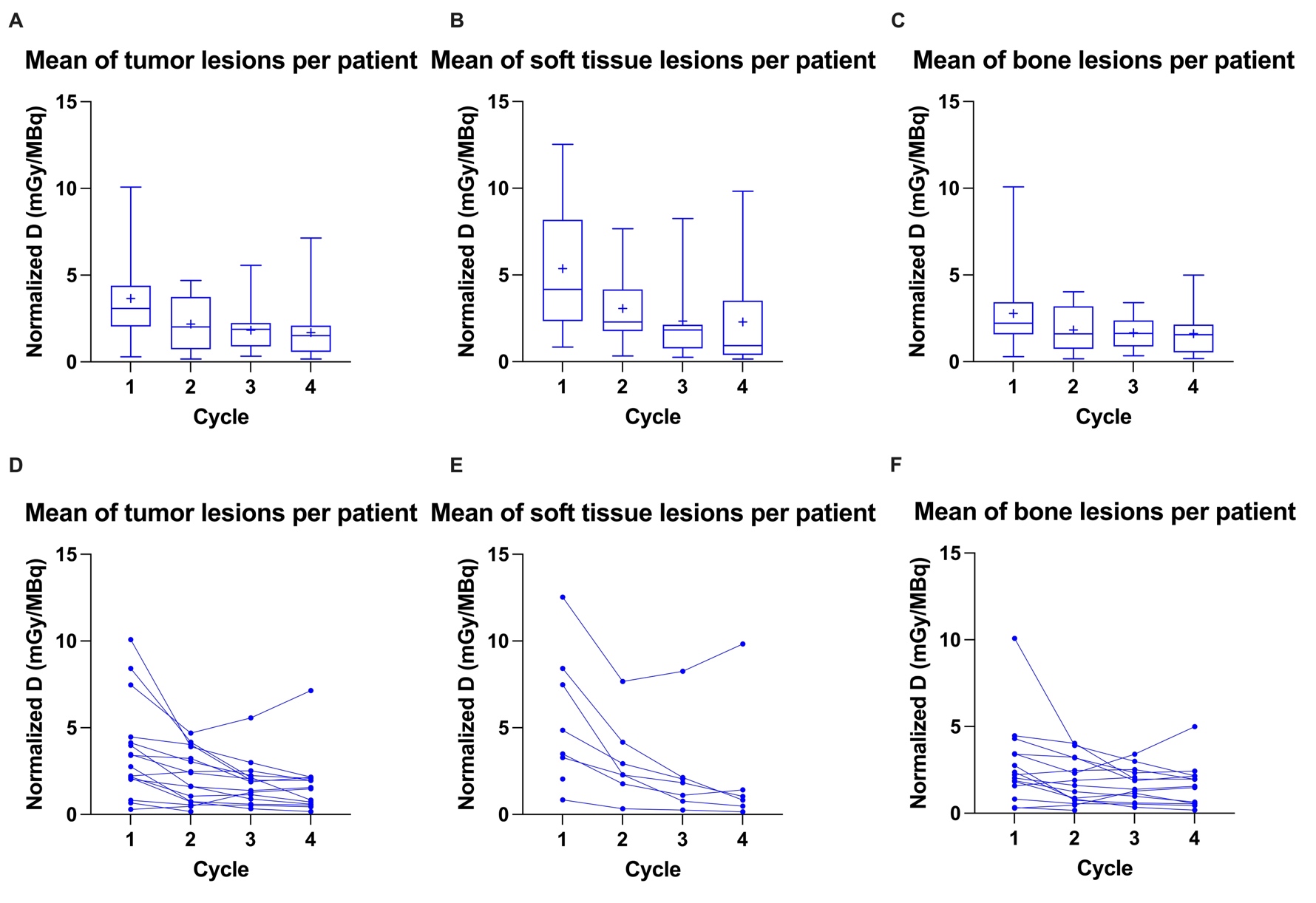


*Supplementary Figure 1: Box-Whisker-Plots showing the quartiles, the 5^th^ and 95^th^ percentiles (whiskers) and the mean (+) of the mean normalized absorbed doses (Normalized D) of tumor lesions per patient (A), soft tissue lesions per patient (B) and bone lesions per patient (C). The course of values over the first four treatment cycles is shown for the mean of all tumor lesions per patient (D), the mean of soft tissue lesions per patient (E) and the mean of bone lesions per patient (F).*

*Supplementary Table 1: Bland-Altman analysis for normalized absorbed doses comparing RM to SM1 using single time-point imaging at 2-4, 24, 48 and 72-168 h p.i. for treatment cycles 2 to 4 for kidneys, parotid glands, and submandibular glands. Data is presented as mean bias (%) ± 1.96*SD (min to max of 95% limits of agreement).*

|  | Kidneys | | | | Parotid glands | | | | Submandibular glands | | | |
| --- | --- | --- | --- | --- | --- | --- | --- | --- | --- | --- | --- | --- |
| Cycles | 2-4 h | 24 h | 48 h | 72-168 h | 2-4 h | 24 h | 48 h | 72-168 h | 2-4 h | 24 h | 48 h | 72-168 h |
| 2 | \| -9.91 ± 34.7 \| \| --- \| \| (-44.6-24.7) \| \|  \| | \| 2.53 ± 23.9 \| \| --- \| \|  \| \|  \| \| (-21.4-26.4) \| \|  \| | \| -2.59 ± 27.9 \| \| --- \| \|  \| \|  \| \| (-30.5-25.3) \| \|  \| | \| -7.66 ± 55.7 \| \| --- \| \|  \| \|  \| \| (-63.3-48.0) \| \|  \| | \| -9.94 ± 70.9 \| \| --- \| \|  \| \|  \| \| (-80.9-61.0) \| \|  \| | \| 16.0 ± 33.1 \| \| --- \| \|  \| \|  \| \| (-17.1-49.1) \| \|  \| | \| 0.62 ± 26.7 \| \| --- \| \|  \| \|  \| \| -26.1-27.3 \| \|  \| | \| 3.66 ± 42.0 \| \| --- \| \|  \| \|  \| \| (-38.4-45.7) \| \|  \| | \| -1.09 ± 45.3 \| \| --- \| \|  \| \|  \| \| (-46.4-44.2) \| \|  \| | \| 6.43 ± 36.9 \| \| --- \| \|  \| \|  \| \| (-30.4-43.3) \| \|  \| | \| -7.87 ± 42.5 \| \| --- \| \|  \| \|  \| \| (-50.4-34.6) \| \|  \| | \| 0.38 ± 47.0 \| \| --- \| \|  \| \|  \| \| (-46.6-47.4) \| \|  \| |
| 3 | \| -8.36 ± 45.4  (-53.8-37.1) \| \| --- \| \|  \| \|  \| \|  \| \|  \| | \| 6.99 ± 18.3 \| \| --- \| \|  \| \|  \| \| (-11.3-25.3) \| \|  \| | \| -0.32 ± 25.0 \| \| --- \| \|  \| \|  \| \| (-25.3-24.7) \| \|  \| | \| -3.09 ± 47.6 \| \| --- \| \|  \| \|  \| \| (-50.6-44.5) \| \|  \| | \| -7.82 ± 79.9 \| \| --- \| \|  \| \|  \| \| (-87.8-72.1) \| \|  \| | \| 11.1 ± 33.4 \| \| --- \| \|  \| \|  \| \| (-22.4- 44.5) \| \|  \| | \| -6.15 ± 41.9 \| \| --- \| \|  \| \|  \| \| (-48.0-35.7) \| \|  \| | \| -11.0 ± 49.9 \| \| --- \| \|  \| \|  \| \| (-60.9-38.9) \| \|  \| | \| 0.42 ± 52.7 \| \| --- \| \|  \| \|  \| \| (-52.3-53.1) \| \|  \| | \| 8.13 ± 29.6 \| \| --- \| \|  \| \|  \| \| (-21.5-37.8) \| \|  \| | \| -5.82 ± 29.7 \| \| --- \| \|  \| \|  \| \| (-35.6-23.9) \| \|  \| | \| -11.6 ± 51.4 \| \| --- \| \|  \| \|  \| \| (-62.9-39.8) \| \|  \| |
| 4 | \| 1.27 ± 49.6 \| \| --- \| \|  \| \|  \| \| (-48.3-50.8) \| \|  \| | \| 7.26 ± 31.8 \| \| --- \| \|  \| \|  \| \| (-24.5-39.0) \| \|  \| | \| -0.87 ± 25.7 \| \| --- \| \|  \| \|  \| \| (-26.6-24.9) \| \|  \| | \| -4.70 ± 56.4 \| \| --- \| \|  \| \|  \| \| (-61.1-51.7) \| \|  \| | \| -10.2 ± 75.9 \| \| --- \| \|  \| \|  \| \| (-86.1-65.7) \| \|  \| | \| 7.98 ± 26.6 \| \| --- \| \|  \| \|  \| \| (-18.7-34.6) \| \|  \| | \| -0.43 ± 35.8 \| \| --- \| \|  \| \|  \| \| (-36.2-35.3) \| \|  \| | \| -6.57 ± 52.6 \| \| --- \| \|  \| \|  \| \| (-59.1-46.0) \| \|  \| \|  \| | \| 8.80 ± 67.0 \| \| --- \| \|  \| \|  \| \| (-58.2-75.8) \| \|  \| | \| 12.2 ± 30.9 \| \| --- \| \|  \| \|  \| \| (-18.7-43.1) \| \|  \| | \| -0.73 ± 39.6 \| \| --- \| \|  \| \|  \| \| (-40.3-38.9) \| \|  \| | \| -10.0 ± 40.5 \| \| --- \| \|  \| \|  \| \| (-50.5-30.5) \| \|  \| |

*Supplementary Table 2: Bland-Altman analysis for normalized absorbed doses comparing RM to SM1 using single time-point imaging at 2-4, 24, 48 and 72-168 h p.i. for treatment cycles 2 to 4 for all individual tumor lesions and as mean of tumor lesions per patient. Data is presented as mean bias (%) ± 1.96*SD (min to max of 95% limits of agreement).*

|  | Individual tumor lesions | | | | Mean of tumor lesions per patient | | | |
| --- | --- | --- | --- | --- | --- | --- | --- | --- |
| Cycles | 2-4 h | 24 h | 48 h | 72-168 h | 2-4 h | 24 h | 48 h | 72-168 h |
| 2 | \| 6.80 ± 55.7 \| \| --- \| \| (48.9-62.5) \| \|  \| | \| 3.91 ± 39.1 \| \| --- \| \|  \| \|  \| \| (-35.2-43.0) \| \|  \| | \| 5.61 ± 48.8 \| \| --- \| \|  \| \|  \| \| (-30.5-25.3) \| \|  \| | \| -1.33 ± 32.6 \| \| --- \| \|  \| \|  \| \| (-33.9-31.3) \| \|  \| | \| 6.16 ± 47.6 \| \| --- \| \|  \| \|  \| \| (-41.5-53.8) \| \|  \| | \| 4.85 ± 29.2 \| \| --- \| \|  \| \|  \| \| (-24.4-34.0) \| \|  \| | \| 7.31 ± 33.2 \| \| --- \| \|  \| \|  \| \| -25.9-40.6 \| \|  \| | \| -0.44 ± 29.7 \| \| --- \| \|  \| \|  \| \| (-30.2-29.3) \| \|  \| |
| 3 | \| 12.6 ± 65.1  (-52.4-77.7) \| \| --- \| \|  \| \|  \| \|  \| \|  \| | \| 3.32 ± 44.6 \| \| --- \| \|  \| \|  \| \| (-41.3-47.9) \| \|  \| | \| 0.17 ± 38.7 \| \| --- \| \|  \| \|  \| \| (-38.6-38.9) \| \|  \| | \| -8.58 ± 28.2 \| \| --- \| \|  \| \|  \| \| (-36.8-19.6) \| \|  \| | \| 13.7 ± 52.3 \| \| --- \| \|  \| \|  \| \| (-38.6-66.0) \| \|  \| | \| 5.53 ± 35.4 \| \| --- \| \|  \| \|  \| \| (-29.8-40.9) \| \|  \| | \| 2.46 ± 30.8 \| \| --- \| \|  \| \|  \| \| (-28.3-33.2) \| \|  \| | \| -8.22 ± 21.2 \| \| --- \| \|  \| \|  \| \| (-29.4-13.0) \| \|  \| |
| 4 | \| 10.5 ± 75.9 \| \| --- \| \|  \| \|  \| \| (-65.4-86.4) \| \|  \| | \| 1.01 ± 39.2 \| \| --- \| \|  \| \|  \| \| (-38.2-40.2) \| \|  \| | \| -1.76 ± 34.0 \| \| --- \| \|  \| \|  \| \| (-35.8-32.3) \| \|  \| | \| -9.31 ± 30.6 \| \| --- \| \|  \| \|  \| \| (-39.9-21.3) \| \|  \| | \| 12.4 ± 51.1 \| \| --- \| \|  \| \|  \| \| (-38.7-63.4) \| \|  \| | \| 2.15 ± 30.2 \| \| --- \| \|  \| \|  \| \| (-27.9-32.2) \| \|  \| | \| 0.99 ± 26.7 \| \| --- \| \|  \| \|  \| \| (-25.7-27.7) \| \|  \| | \| -7.85 ± 19.5 \| \| --- \| \|  \| \|  \| \| (-27.3-11.6) \| \|  \| \|  \| |


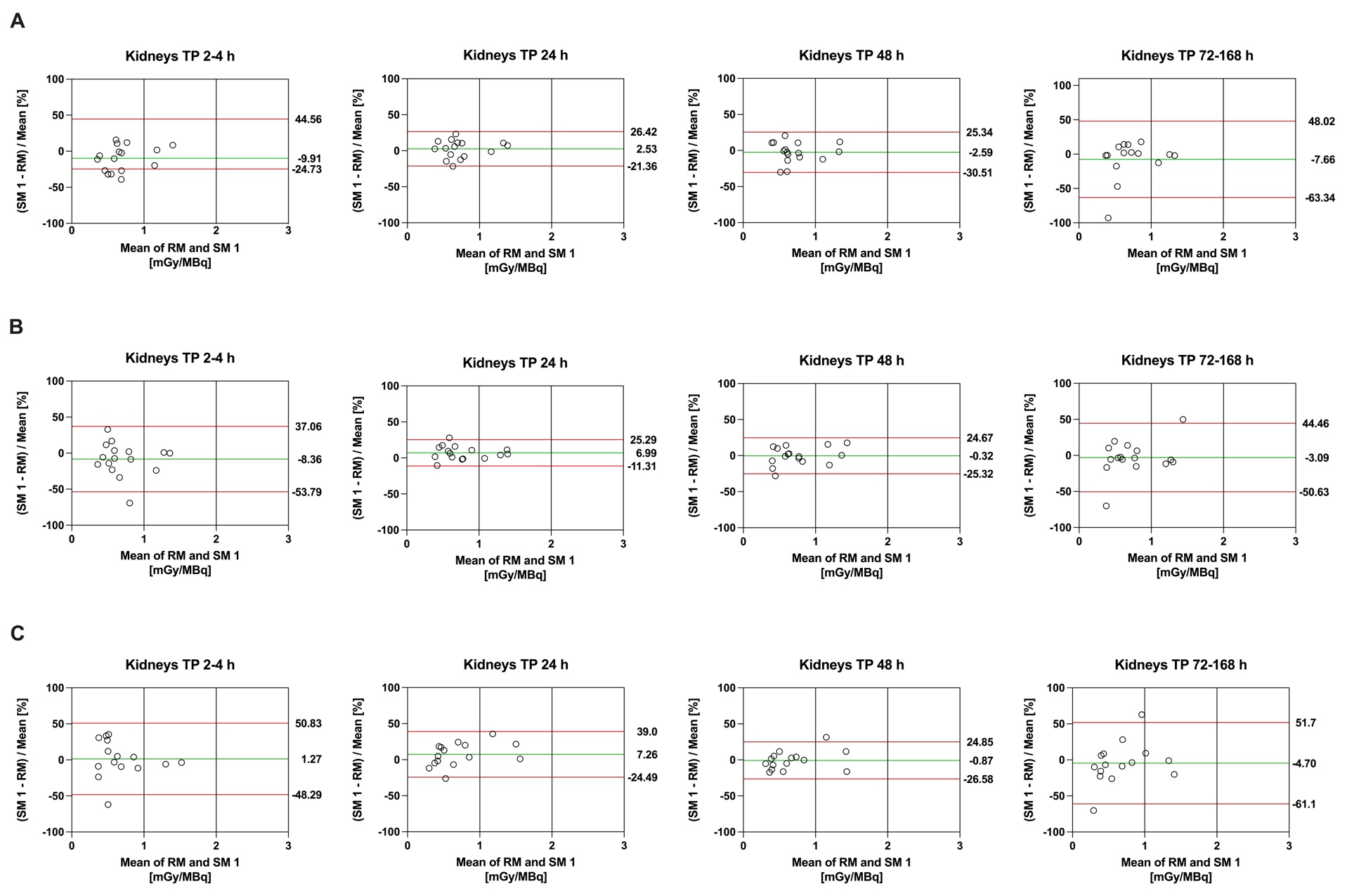


*Supplementary Figure 2: Bland-Altman for normalized absorbed doses plots comparing RM to SM1 using single time-point (TP) imaging at 2-4, 24, 48 and 72-168 h p.i. for kidneys at treatment cycle two (A), three (B) and four (C). The green line represents the mean bias between the two methods, the red lines show the 95% limits of agreement.*


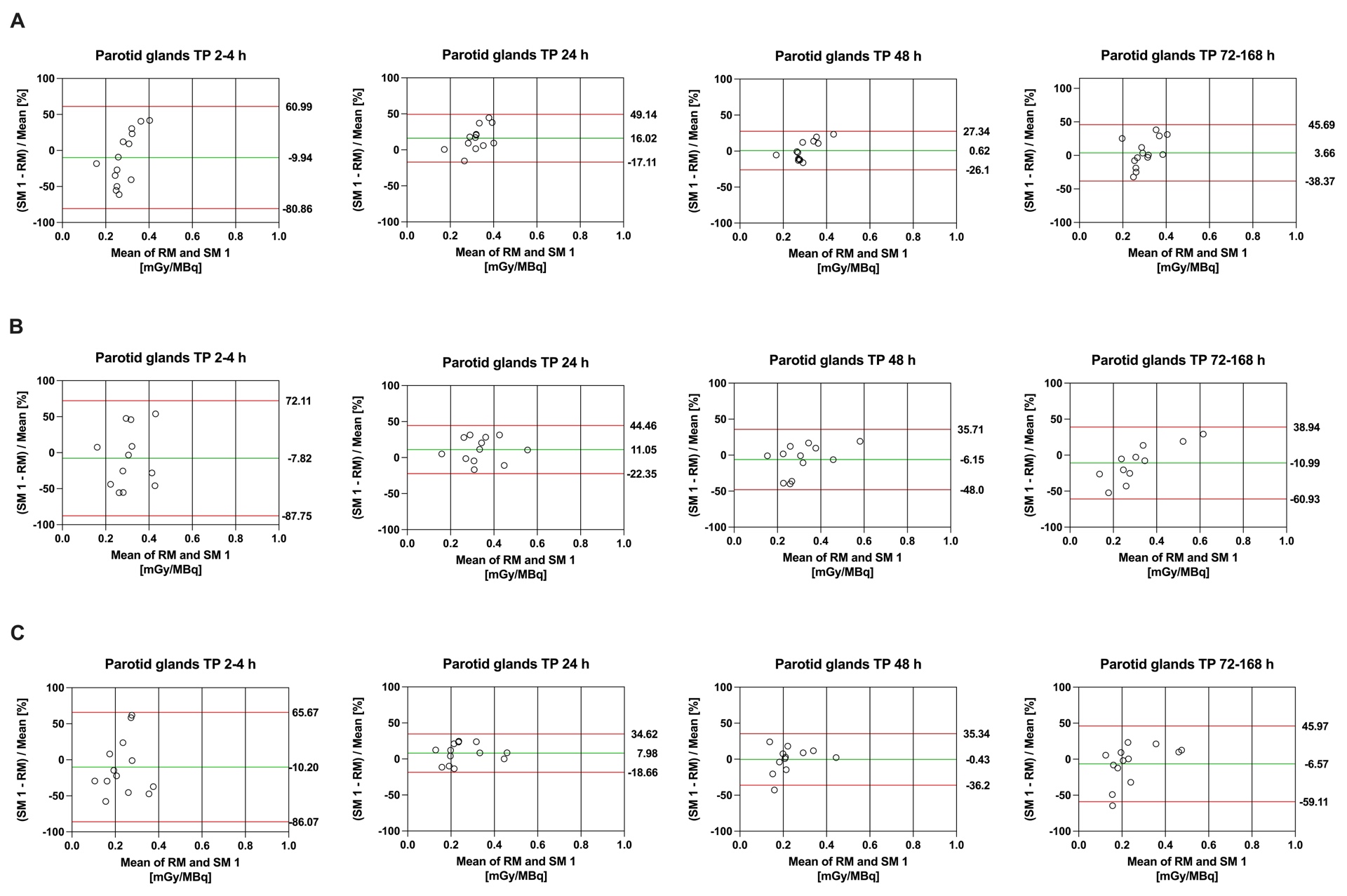


*Supplementary Figure 3: Bland-Altman plots for normalized absorbed doses comparing RM to SM1 using single time-point (TP) imaging at 2-4, 24, 48 and 72-168 h p.i. for parotid glands at treatment cycle two (A), three (B) and four (C). The green line represents the mean bias between the two methods, the red lines show the 95% limits of agreement.*


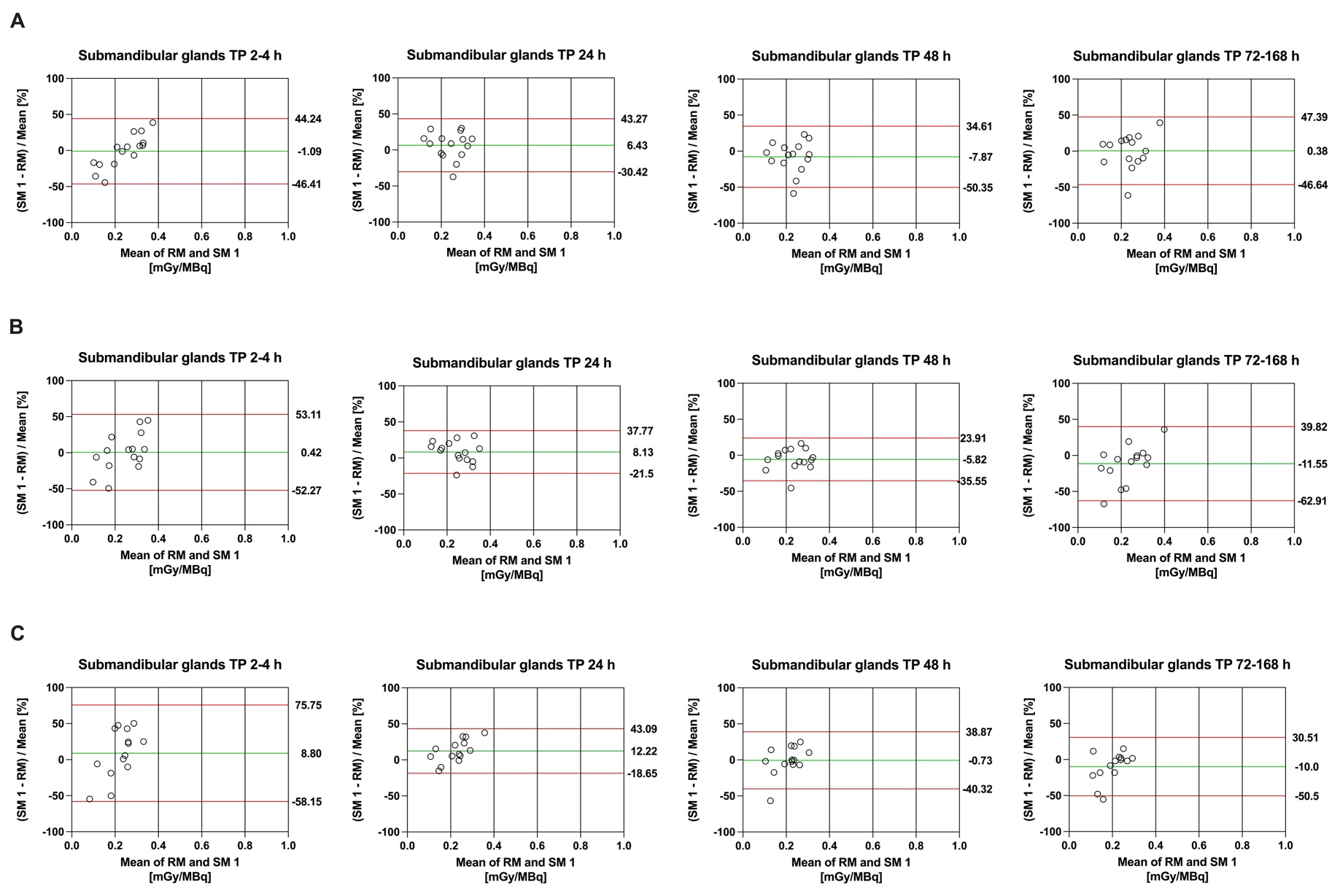


*Supplementary Figure 4: Bland-Altman for normalized absorbed doses plots comparing RM to SM1 using single time-point (TP) imaging at 2-4, 24, 48 and 72-168 h p.i. for submandibular glands at treatment cycle two (A), three (B) and four (C). The green line represents the mean bias between the two methods, the red lines show the 95% limits of agreement.*

*
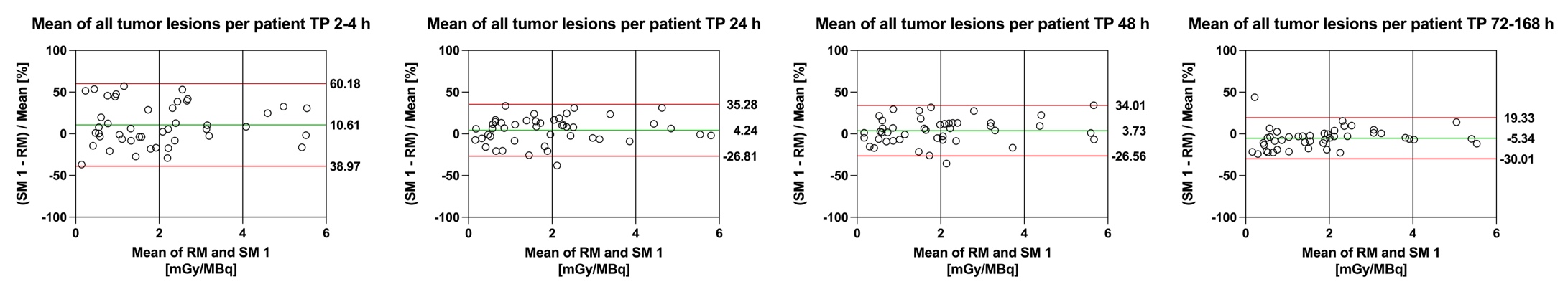
*

*Supplementary Figure 5: Bland-Altman plots for normalized absorbed doses comparing RM to SM1 using single time-point (TP) imaging at 2-4, 24, 48 and 72-168 h p.i. combined for treatment cycles 2 to 4 for the mean of tumor lesions per patient. The green line represents the mean bias between the two methods, the red lines show the 95% limits of agreement.*


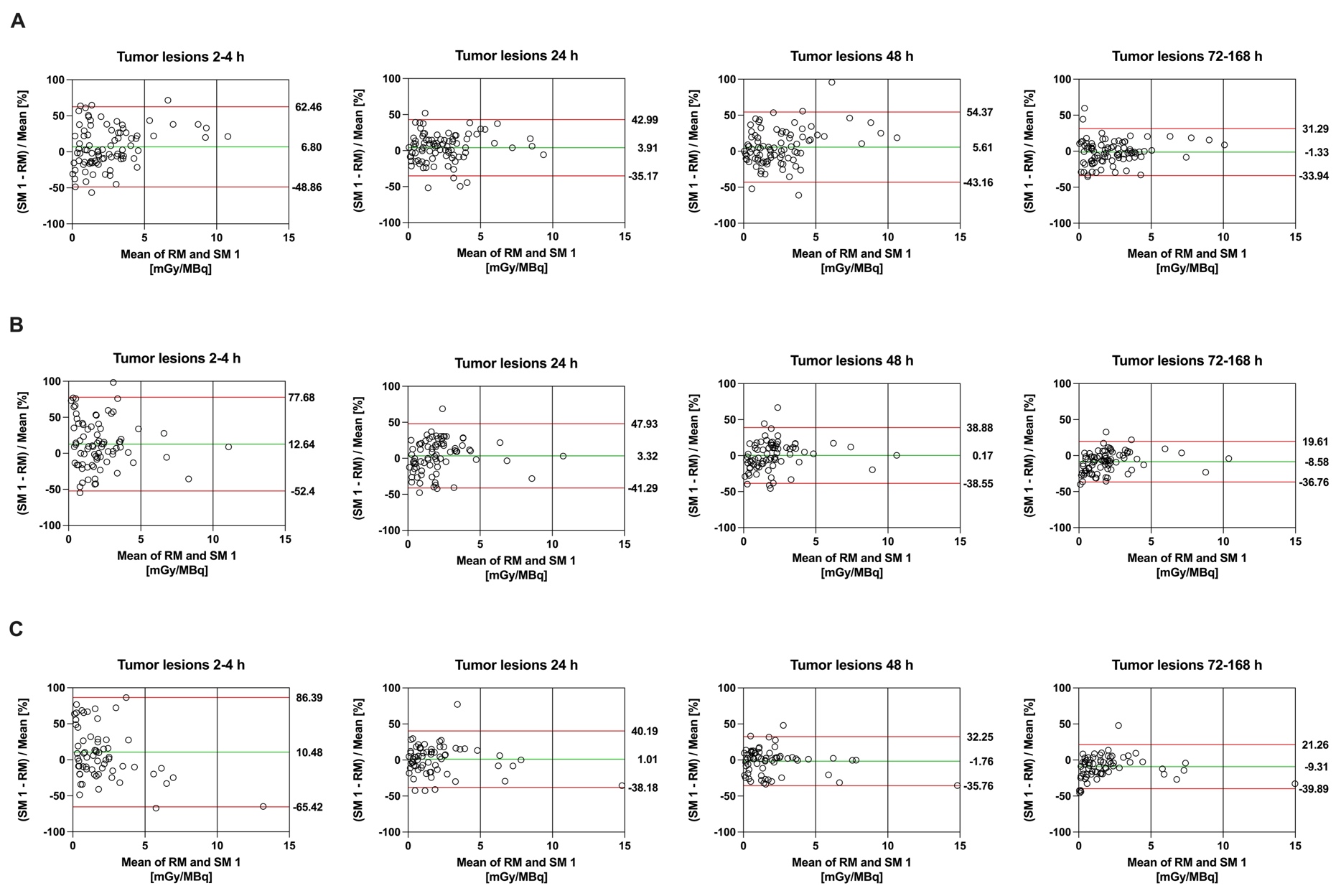


*Supplementary Figure 6: Bland-Altman plots for normalized absorbed doses comparing RM to SM1 using single time-point (TP) imaging at 2-4, 24, 48 and 72-168 h p.i. for individual tumor lesions at treatment cycle two (A), three (B) and four (C). The green line represents the mean bias between the two methods, the red lines show the 95% limits of agreement.*


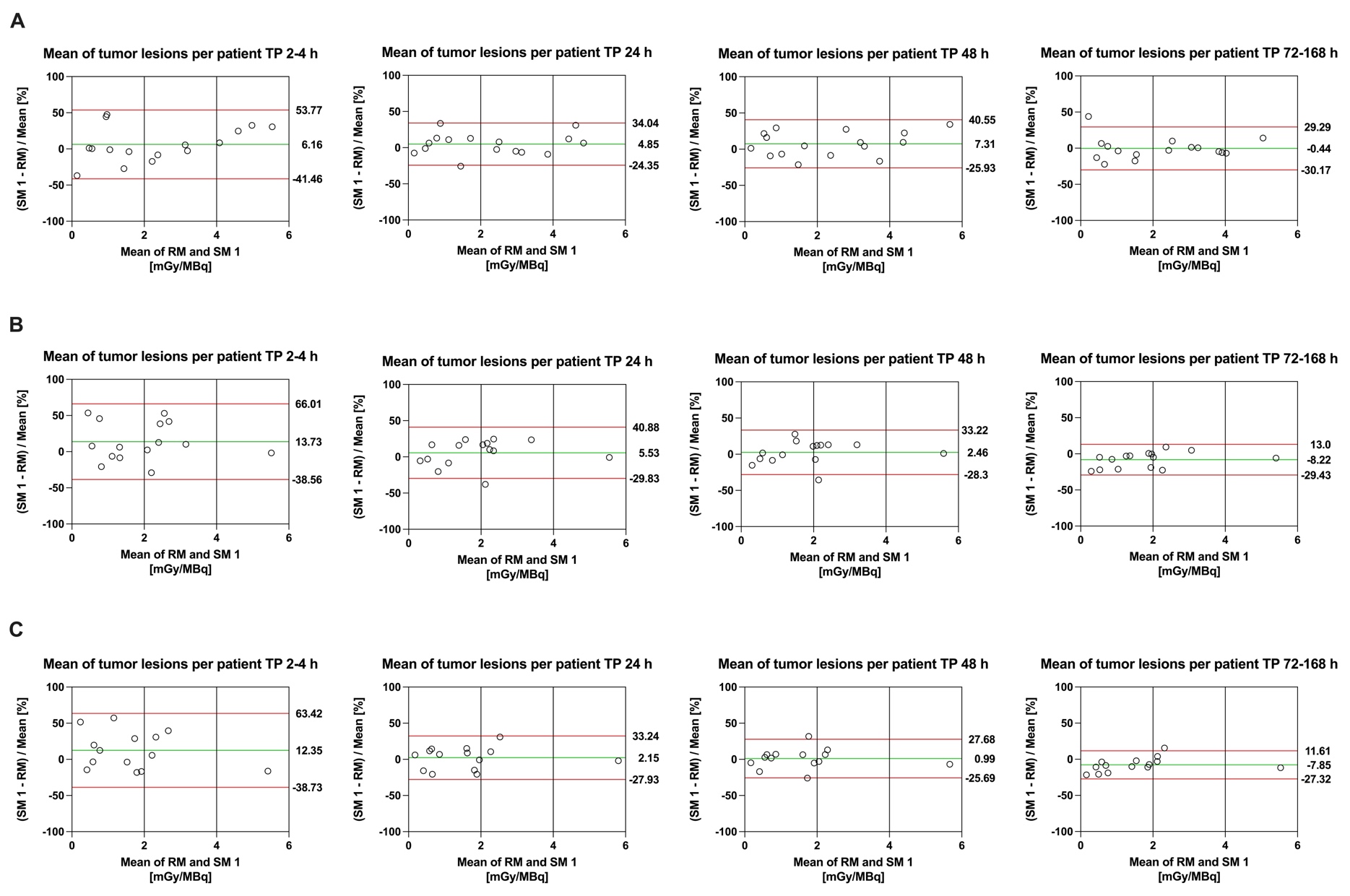


*Supplementary Figure 7: Bland-Altman plots for normalized absorbed doses comparing RM to SM1 using single time-point (TP) imaging at 2-4, 24, 48 and 72-168 h p.i. for the mean of tumor lesions per patient at treatment cycle two (A), three (B) and four (C). The green line represents the mean bias between the two methods, the red lines show the 95% limits of agreement.*
